# MultivariateSystem Identification of Differential Drive Robot: Comparison Between State-Space and LSTM-Based Models

**DOI:** 10.3390/s25185821

**Published:** 2025-09-18

**Authors:** Diego Guffanti, Wilson Pavon

**Affiliations:** Universidad UTE, Av. Mariscal Sucre, Quito 170129, Ecuador; wilson.pavon@ute.edu.ec

**Keywords:** mobile robots, differential drive robots, identification, modeling, space-state, recurrent neural networks, long short-term memory

## Abstract

Modeling mobile robots is crucial to odometry estimation, control design, and navigation. Classical state-space models (SSMs) have traditionally been used for system identification, while recent advances in deep learning, such as Long Short-Term Memory (LSTM) networks, capture complex nonlinear dependencies. However, few direct comparisons exist between these paradigms. This paper compares two multivariate modeling approaches for a differential drive robot: a classical SSM and an LSTM-based recurrent neural network. Both models predict the robot’s linear (*v*) and angular (ω) velocities using experimental data from a five-minute navigation sequence. Performance is evaluated in terms of prediction accuracy, odometry estimation, and computational efficiency, with ground-truth odometry obtained via a SLAM-based method in ROS2. Each model was tuned for fair comparison: order selection for the SSM and hyperparameter search for the LSTM. Results show that the best SSM is a second-order model, while the LSTM used seven layers, 30 neurons, and 20-sample sliding windows. The LSTM achieved a FIT of 93.10% for *v* and 90.95% for ω, with an odometry RMSE of 1.09 m and 0.23 rad, whereas the SSM outperformed it with FIT values of 94.70% and 91.71% and lower RMSE (0.85 m, 0.17 rad). The SSM was also more resource-efficient (0.00257 ms and 1.03 bytes per step) compared to the LSTM (0.0342 ms and 20.49 bytes). The results suggest that SSMs remain a strong option for accurate odometry with low computational demand while encouraging the exploration of hybrid models to improve robustness in complex environments. At the same time, LSTM models demonstrated flexibility through hyperparameter tuning, highlighting their potential for further accuracy improvements with refined configurations.

## 1. Introduction

The modeling of mobile robots is a crucial activity in robotics, as it facilitates the prediction of motion, design, and planning for control and navigation. Among the different types of mobile robots, differential drive robots are the most common configuration because they are easy to design and move around. Despite this, the accurate modeling of their dynamics is a challenge, as it involves expressing the non-linear relationship between control inputs and the robot’s motion [1,2].

In MIMO (Multiple Input, Multiple Output) systems, the interaction between different state variables must be properly accounted for. In the case of a differential drive robot, the linear velocity (*v*) and the angular velocity (*w*) are usually coupled, making it essential to use multivariate modeling approaches for system identification. Traditional system identification methods, such as state-space models (SSM), provide a structured representation of the dynamics of MIMO systems, explicitly modeling the interaction between multiple control inputs and state variables based on physical considerations and experimental evidence [3]. These models have been effective in estimating dynamic parameters and designing controllers for mobile robots [4].

Modern developments in machine learning methods have brought novel data-driven solutions, like deep learning models, capable of learning intricate system dynamics from large sets of data in an end-to-end manner [5]. Contrary to traditional identification techniques, deep learning models can learn multivariate output–input relationships directly from data. Recurrent neural networks (RNNs), particularly Long Short-Term Memory (LSTM) networks, have shown promising results in modeling dynamical systems [6]. These models can capture temporal dependencies in the data, making them ideal for learning the dynamic response of robotic systems [7]. Unlike traditional parametric models, neural networks do not require explicit system equations as the majority are end-to-end approaches [5], making them an attractive alternative for system identification, especially in scenarios with unmodeled dynamics or complex disturbances [8].

Deep learning approaches to robot modeling have been examined in several research papers. An example is the exploration of the use of deep-recurrent models in autonomous robot navigation to improve the accuracy of odometry and trajectory tracking [9]. Hybrid approaches toward the fusion of data-driven learning and physics-based models have also been reported to be effective in terms of improved robustness and generalization in robotic tasks [9]. However, these methods often have many problems, such as high demand for training data, overfitting, lack of explainability, and the requirement of extensive computational resources, which may limit their applicability in real-time control. Despite these limitations, authors such as Carreón et al. (2021) proposed a neural network-based algorithm to extract dynamic parameters from robots, demonstrating the effectiveness of data-driven models even with incomplete motion data, highlighting their potential for real-world robotic applications [10].

Despite the promise of classical state-space models and deep learning methods, few studies have directly compared these two paradigms under the same conditions, especially in the context of multivariate identification of differential drive robots. The novelty of this work lies in addressing that gap. First, we provide a systematic, side-by-side evaluation of SSMs and LSTM for identifying both linear and angular velocities (v,ω) in a multivariate framework, using experimental data from a differential drive robot. Second, we explore how different SSM model orders and hyperparameter configurations of LSTM (e.g., window size, number of neurons, activation functions, optimizers) affect modeling accuracy, highlighting current trade-offs. Third, we assess not only prediction accuracy, but also computational efficiency, an aspect crucial for real-time robotic applications, thereby offering insights into the practicality of deploying each method. Finally, our study bridges traditional and modern approaches, providing guidance on when classical models may remain preferable and when LSTM architectures are more advantageous, thereby providing value for researchers and practitioners in robotic system identification.

The outline of this paper is organized as follows. Section 2 presents a state-of-the-art review of current methods applied to the modeling and system identification of mobile robot systems. Section 3 is dedicated to the methodology. This section explains the mobile robotic platform used in this study, the data collection details, and the identification process followed using the SSM and LSTM approaches. Section 4 addresses the comparison of both approaches and the accuracy achieved in each. Section 5 discusses the results of this study by comparing them with other current approaches. Finally, Section 6 concludes this study and outlines future work.

## 2. Review of the State-of-the-Art

The modeling and identification of mobile robots, specifically differential drive robots, have been extensively researched on the basis of their importance in control and navigation. Classical models like SSM have been extensively employed based on their efficacy in determining structured and interpretable dynamic system representations. SSM models the relation of control input to the subsequent motion of the robot and is thus beneficial in the estimation of velocity, the estimation of odometry, and the design of the controller. Khalil and Dombre (2002), for example, showcased how SSM successfully models robot dynamics to enhance velocity estimation and trajectory tracking, an area of significant use in mobile robotics [11].

Deep learning methods, specifically LSTM networks, have in recent years attracted attention in system identification tasks [6]. The time dependencies in the LSTM models make them suitable for the estimation of the velocity and the prediction of the motion of robots over time. Kiwon et al. (2020) evaluated a DNN-MPC system in mobile robot navigation, in which it decreased the maximum positioning error in the *x* direction to 123 mm and in the *y* direction to 32 mm, compared to conventional MPC with errors of 156 mm and 41 mm, respectively [12]. These results highlight the potential of deep learning to improve trajectory tracking accuracy.

Recent work has also shown progress in the application of LSTM in engineering and mobile robotics. For example, Li et al. (2025) developed a 6-DoF nonlinear model for an underactuated underwater vehicle that combines conventional EKF parameter estimation with an LSTM network for time-series prediction of linear and angular velocities, complemented by a nonlinear complementary filter (NECF) to prevent drift in dead-reckoning [13]. Zhang et al. (2025) proposed an adaptive-enhanced time series clustering method (TSCM) within computational mechanics, using advanced clustering to improve reliability analysis and design optimization under time-varying uncertainties [14]. Alcayaga et al. (2025) integrated LSTM into a deep reinforcement learning control policy for skid steer mobile robots navigating challenging, variable-traction terrains, enabling robust trajectory tracking even under partial observability [15]. Additionally, Reginald (2025) introduced a data-driven wheel-slip compensation framework using Gaussian Process Regression with LSTM layers to improve visual-inertial-wheel odometry accuracy under dynamic terrain conditions [16]. This contribution demonstrates the applicability of LSTM-based approaches in engineering contexts, particularly for improving the robustness of navigation systems in ground vehicles operating on irregular or low-traction surfaces.

Although LSTM-based models are beneficial, they are sensitive to the size of the data set, hyperparameters, and noise in real-world environments [5]. Zheng et al. (2022) adapted an LSTM-based system using 1000 sets of samples and reached near-zero test error under controlled conditions [17]. However, real-world applications are typically characterized by larger error levels due to unmodeled behavior and sequential error compounding. Zheng et al. (2022) studied the impacts of communication delay and packet loss on trajectory tracking and found that a 10% packet loss had a significant impact on system performance [17]. Because LSTM models are based so much on sequential data, the networks are potentially susceptible to compounding errors in long sequences.

Another distinction of LSTM-based odometry models is their structure. Farina et al. (2023) developed an LSTM-based odometry model in which two separate neural networks were learned to estimate the linear and angular velocities independently [18]. The model had an accuracy of 86.57% and an RMSE of 0.102 m/s for linear velocity (*v*) and 0.179 rad/s for angular velocity (ω), compared to traditional odometry, where the accuracy was 80.2% and the RMSE is higher (0.123 m / s for *v* and 0.200 rad/s for ω). Even with this model showing improvements over the traditional methods, it did not investigate the benefits of a multivariable framework where the two velocities are estimated simultaneously.

The study of deep learning in the identification of drive robots is still evolving, and the choice between traditional and learning-based methods depends on the application requirements. Previous research has reported that deep learning-based controllers typically have stabilization times around 2.3 s, with maximum angular errors below 6 degrees [12]. These insights suggest that, while LSTM models offer flexibility in capturing nonlinear behaviors, traditional approaches like SSM could remain valuable for structured robotic tasks.

Comparison of multivariate system identification techniques presents a promising direction for improving velocity estimation and odometry. Unlike architectures that estimate *v* and ω separately, a multivariate approach allows capturing the interdependencies between linear and angular motion, potentially leading to more accurate and stable robotic control systems. This paper explores the comparison between classical system identification models based in state-space (SSM) and a modern approach based on LSTM neural networks, evaluating their effectiveness in improving system performance, particularly in the precise estimation of velocities and odometry data.

## 3. Methodology

The MIMO approaches are suitable for modeling differential drive robots when it is observed that the dynamics of *v* and ω are not completely independent, i.e., that changing *v* also affects ω and vice versa. This can happen in several situations in a differential drive robot, for example, if the distance between wheels is not perfectly uniform or there is a small error in the implementation of the motor control, a coupling between the two movements may occur. In addition, effects such as inertia or delays in motors make it possible that a change in *v* or ω is not immediately reflected in the expected output, and there is a dynamic interaction between both. The following sections explain the multivariate modeling approaches addressed in this paper, as well as the robotic platform and the experimental data collection for the identification procedure.

### 3.1. Mobile Robotic Platform

**Figure 1:**
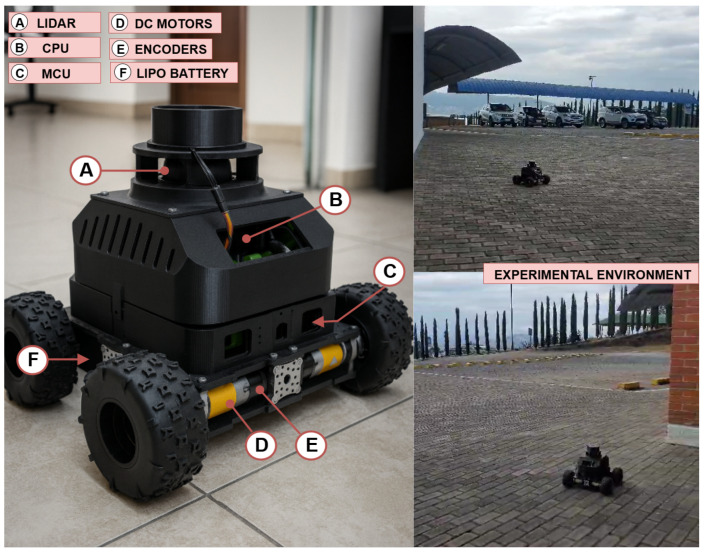
Real robot overview and experimental environment.

This paper analyzes a differential drive robot with 4 wheels driven by 4 encoder-powered motors. The electronics include ROBOCLAW controllers, a LIDAR sensor, an IMU, and an STM32 MCU (STM32-F412ZG) programmed with HAL and C++ libraries. The robot also has an onboard CPU with the Linux Ubuntu 20.04 operating system and the ROS2 framework, specifically the ROS2-Foxy version, which is responsible for sending commands and receiving relevant data. The Navigation (NAV2) and SLAM (Slam Toolbox) packages from ROS2 are also loaded into the system. These packages allow for retrieving an accurate real odometry calculation. The drivers have a built-in velocity controller that can be tuned through a user interface named Basicmicro Motion Studio. The drives communicate with the microcontroller through a 38,400 baud USART serial connection. The microcontroller communicates with the onboard CPU through a serial port using a USB cable. The information about the actual velocity of the robot comes from the robot’s own electronics, which includes the encoders and the IMU sensor. The entire system has a sampling time of 0.0202 s, which is approximately 50 Hz. Additional components of the mobile robotic platform can be found in Figure 1.

### 3.2. Experimental Data Collection

To train the SSM model and the LSTM model, a five-minute experiment was performed in which the robot was freely driven over a rough concrete surface with high friction and irregular texture, chosen to minimize wheel slippage during motion. The experimental environment, including the robot operating on this terrain, can be observed in Figure 1. A total of 16,292 samples were collected in a csv file. The data contains the time stamp, the linear velocity input command ($v_{ref}$), the angular velocity input command ($\omega_{ref}$), the actual linear velocity of the robot (*v*), and its actual angular velocity (ω). In order to compare the modeling approaches discussed in this paper, 2 additional experiments with free trajectories with an approximate navigation duration of about 30 s were performed. During this time, in addition to collecting the linear and angular velocity information of the robot, the ROS2 SLAM package was also used to collect information on the odometry of the robot. This included the positions *x* and *y*, and the yaw angle (θ) of the robot’s orientation. All experiments were performed with velocity input commands ($v_{ref}$, $\omega_{ref}$) ranging from 1.5 m/s and 2.0 rad/s respectively. The data collected was merged into data objects and processed using Matlab R2023b^®^.

### 3.3. Evaluation Metrics

The performance of both models was quantitatively assessed using the Goodness of Fit (FIT) and the Root Mean Squared Error (RMSE). FIT and RMSE were calculated for both the linear velocity (*v*) and the angular velocity (ω), as well as the odometry trajectories. The metrics are defined as follows:

(1) $$\mathrm{RMSE}=\sqrt{\frac{1}{M}\sum_{i=1}^{M}{\left(y\left(i\right)-\hat{y}\left(i\right)\right)}^{2}}$$

(2) $$\mathrm{FIT}\;(\%)=100\cdot \left(1-\frac{∥y-\hat{y}∥}{∥y-\bar{y}∥}\right)$$

where y(i) is the measured output, $\hat{y}\left(i\right)$ is the model output and *M* is the number of samples. For trajectory evaluation, the RMSE was calculated using the Euclidean distance between the simulated and measured positions.

### 3.4. State-Space Model (SSM)

A MIMO modeling based on state-space can describe the system in terms of its internal states. It uses state matrices to describe the dynamics of the system. The general state-space equation used to represent the system dynamics is the following Equations (3) and (4):

(3) $$\dot{x}=Ax+Bu$$

(4) y=Cx+Du

where *x* is the state vector, *u* is the input vector ($v_{ref}$, $\omega_{ref}$), *y* is the output vector (*v*, ω), *A* is the system dynamics matrix, *B* is the input matrix relating the input to the states, *C* is the output matrix relating the states to the outputs, and *D* is the direct transmission matrix.

Equations (3) and (4) are derived from the general dynamic modeling of a differential drive robot. In this formulation, the state vector *x* represents the internal variables that describe the motion of the robot, while the input vector $u=(v_{\mathrm{ref}},\omega_{\mathrm{ref}})$ contains the commanded linear and angular velocities. The output vector y=(v,ω) corresponds to the measured velocities obtained from the robot during the experiments. The matrices *A*, *B*, *C*, and *D* are identified experimentally: *A* captures the intrinsic dynamics of the robot, *B* links the control inputs to the state evolution, *C* maps the internal states to the measurable outputs, and *D* accounts for any direct transmission between inputs and outputs. This formulation follows the standard multivariable system identification framework, but is here adapted to describe the coupled dynamics of linear and angular velocities in differential robots.

The order of a state-space model represents the number of internal states that describe the dynamics of the system. In our case, since two internal variables will be modeled (*v*, ω), the minimum number of states should be 2. However, higher-order models can improve the fit in some cases. The addition of state variables can be useful to represent dynamics that cannot be adequately captured with only two states, such as the acceleration of *v* or ω, or additional dynamics related to the motors or control. For this reason, during the identification of the robot’s dynamics, different orders were tested in the state-space equation. Each order obeys different motivations, which are detailed below:

- Order 2. This is the minimum reasonable order for this modeling approach, which can capture the relationships between the input velocity ($v_{ref}$, $\omega_{ref}$) and the actual velocities (*v*, ω). It can also include some effect of inertia and delay in response.
- Order 3. This is a more detailed model that may capture accelerations and decelerations of the robot. It could be useful if the robot has a slower dynamic or if there are mechanical delays in its response.
- Order 4. This can capture additional effects such as friction, sliding, or external disturbances, but could be more accurate. However, the model becomes more complex and less interpretable.
- Order 5. This is a highly detailed model that could capture more complex nonlinear dynamics. This is rarely used unless the robot has complicated dynamics, as it can over-fit the data.

Table 1 summarizes the results of this experimentation. The analysis includes a comparison of Goodness of Fit (FIT) and Root Mean Squared Error (RMSE) between orders. The FIT score measures how well the predicted output matches the actual response of the system, expressed as a percentage. A higher FIT value indicates a better agreement between the model and the actual response. RMSE, on the other hand, quantifies the average deviation between predicted and actual response, where lower RMSE values indicate higher predictive accuracy. These metrics allow for quantitative comparison of different order configurations in the SSM approach. When analyzing Table 1 the comparison between order 2 and order 4 reveals a difference in FIT for *v* and ω lower than 0.5%, being better for the higher order. However, increasing the order of the system should justify a significant improvement in FIT, which is not experienced here. On the other hand, order 5 exhibits a worse FIT at ω, where the FIT drops to 85.23%, which suggests either overfitting or that the model captures noise rather than the true dynamics of the robot. Although higher orders, such as 3 and 4, demonstrated slight improvements in FIT, these improvements were minimal. Since a simpler model is preferable for interpretability and robustness, this order was chosen for the final analysis in the SSM approach.

From this second-order state-space, the following matrices were obtained:

(5) $$\begin{array}{cc} A & =\left[\begin{array}{cc} -7.381 & -0.3359\\ 1.104 & -13.31 \end{array}\right] \end{array}$$

(6) $$\begin{array}{cc} B & =\left[\begin{array}{cc} 0.6526 & 0.03428\\ -0.04713 & 0.9911 \end{array}\right] \end{array}$$

(7) $$\begin{array}{cc} C & =\left[\begin{array}{cc} 11.37 & -0.1933\\ -0.5125 & 14.40 \end{array}\right] \end{array}$$

(8) $$\begin{array}{cc} D & =\left[\begin{array}{cc} 0 & 0\\ 0 & 0 \end{array}\right] \end{array}$$

The identified state-space model describes the dynamic relationship between the reference velocities ($v_{ref}$, $\omega_{ref}$) and the actual velocities (*v*, ω) of the robot. The negative diagonal entries in matrix *A* (−7.381, −13.31) represent dissipative effects, mainly associated with motor damping and mechanical friction, which dominate how quickly the velocities decay in the absence of input. The off-diagonal terms (−0.3359, 1.104) reveal the cross-coupling between linear and angular motion, showing how a command in one channel influences the dynamics of the other. The matrix *B* indicates how the inputs excite the system. The large diagonal elements of *B* (0.6526, 0.9911) reflect the direct effect of $v_{ref}$ on *v* and of $\omega_{ref}$ on ω, while the smaller off-diagonal coefficients of *B* (0.03428, −0.04713) capture secondary effects such as asymmetries in actuation or interactions between wheel dynamics. The matrix *C* provides the mapping from the internal states to the measured outputs, scaling the latent dynamics to the observable velocities. Finally, the zero matrix *D* confirms that there is no direct feed-through from inputs to outputs, which is consistent with the fact that robot dynamics are dominated by inertial and frictional effects rather than instantaneous responses.

For a more direct interpretation of the system response, the state-space model can be expressed in the Laplace domain (s) and transformed into the transfer function form according to the following:

(9) $$G\left(s\right)=C{\left(sI-A\right)}^{-1}B+D$$

where *I* is an identity matrix (2×2). The resulting transfer functions are as follows:

(10) $$\frac{v(s)}{v_{\mathrm{ref}}\left(s\right)}=\frac{7.428s+98.86}{s^{2}+20.69s+98.62}$$

(11) $$\frac{v(s)}{\omega_{\mathrm{ref}}\left(s\right)}=\frac{-1.013s+0.9052}{s^{2}+20.69s+98.62}$$

(12) $$\frac{\omega (s)}{v_{\mathrm{ref}}\left(s\right)}=\frac{0.1981s-0.01966}{s^{2}+20.69s+98.62}$$

(13) $$\frac{\omega (s)}{\omega_{\mathrm{ref}}\left(s\right)}=\frac{14.26s+105.9}{s^{2}+20.69s+98.62}$$

The fact that all transfer functions have the same second-order denominator ($s^{2}+20.69s+98.62$) suggests that the system has a common dynamics that affects both outputs (v(s), ω(s)), regardless of whether the reference inputs are (which is typical in differential robots). This denominator represents the dynamics of a second-order system, which is modeling the dynamic response of the robot.

**Figure 2:**
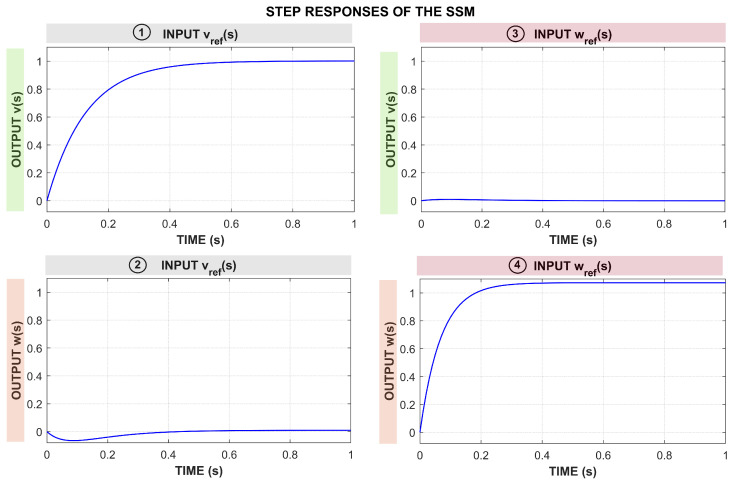
Step responses of the SSM model.

The step responses of the MIMO system shown in Figure 2 describe the dynamic relationships between the reference inputs $v_{\mathrm{ref}}\left(s\right)$ and $\omega_{\mathrm{ref}}\left(s\right)$ and the output velocities v(s) and ω(s). The first subfigure shows that v(s) is mainly influenced by $v_{\mathrm{ref}}\left(s\right)$, with a second-order dynamic response and a positive relationship, where changes in $v_{\mathrm{ref}}\left(s\right)$ lead to significant changes in v(s). The second subfigure indicates the presence of an influence of $v_{\mathrm{ref}}\left(s\right)$ on ω(s), with an inverse relationship. The third subfigure shows that $\omega_{\mathrm{ref}}\left(s\right)$ has a minor effect on v(s), with a smaller magnitude response. Finally, the fourth subfigure reveals that $\omega_{\mathrm{ref}}\left(s\right)$ has a strong positive influence on ω(s), suggesting that changes in $\omega_{\mathrm{ref}}\left(s\right)$ lead to proportional changes in ω(s). In general, the system demonstrates cross-coupling dynamics, where $v_{\mathrm{ref}}\left(s\right)$ affects primarily v(s), and $\omega_{\mathrm{ref}}\left(s\right)$ affects primarily ω(s), while there is also an influence of $v_{\mathrm{ref}}\left(s\right)$ on ω(s).

### 3.5. Recurrent Neural Network Model (LSTM)

In this modeling approach, an LSTM neural network was used to model the dynamic response of the differential drive robot. The data set used to train the LSTM network was the same as that used for the SSM approach explained in the previous subsection. The input data consists of reference commands, denoted as $v_{\mathrm{ref}}$ and $\omega_{\mathrm{ref}}$, together with the corresponding real responses *v* and ω as labels.

To capture temporal dependencies, a sliding-window approach was implemented. We defined a window size WS and constructed input–output pairs ($X_{\mathrm{seq}}$, $Y_{\mathrm{seq}}$) as follows:

(14) $$X_{\mathrm{seq}}\left(j\right)=\left\{\left(v_{\mathrm{ref}}\left(j\right),\omega_{\mathrm{ref}}\left(j\right)\right),\ldots ,\left(v_{\mathrm{ref}}\left(j+WS-1\right),\omega_{\mathrm{ref}}\left(j+WS-1\right)\right)\right\}$$

(15) $$Y_{\mathrm{seq}}\left(j\right)=\left(v\left(j+WS\right),\omega \left(j+WS\right)\right)$$

where $X_{\mathrm{seq}}\left(j\right)$ represents the past WS samples of $v_{\mathrm{ref}}$ and $\omega_{\mathrm{ref}}$, while $Y_{\mathrm{seq}}\left(j\right)$ is the next step prediction target (v,ω). This formulation enables the network to learn the mapping between past reference velocities and future actual velocities. The data set was divided into training sets (75%), validation sets (15%), and testing sets (10%). The training sequences were used to optimize the LSTM-based architecture designed to predict future velocity states.

The general architecture of the LSTM consists of a sequence input layer with two features ($v_{\mathrm{ref}},\omega_{\mathrm{ref}}$), followed by one or more LSTM layers with *N* neurons to capture temporal dependencies. A dropout layer with a rate of 0.2 is included to reduce overfitting, then a fully connected layer with 32 neurons and a non-linear activation function, and finally a fully connected output layer with two neurons generates the predicted values (v,ω). The loss function is computed through a regression layer. The networks were trained using the Adam optimizer with L2 regularization (λ=0.001), mini-batch updates, validation checks, and early stopping based on a patience parameter.

To evaluate the impact of different hyperparameters on performance, several configurations were tested by varying the number of neurons (*N*), window size (WS), batch size (BS), validation frequency (VF), and validation patience (VP), as well as considering different architectures (ARCH), activation functions (ACT), and optimizers (OPT). Beyond the baseline configuration (single LSTM layer with tanh activation and Adam optimizer), three extended variants were explored: (i) a stacked LSTM with two recurrent layers to assess whether additional depth improves temporal modeling; (ii) a dense layer with ReLU activation instead of tanh to assess the effect of nonlinearities; and (iii) the RMSProp optimizer in place of Adam to compare different update rules during training. Table 2 summarizes all the configurations evaluated and their performance metrics in both the validation and the test data sets.

**Figure 3:**
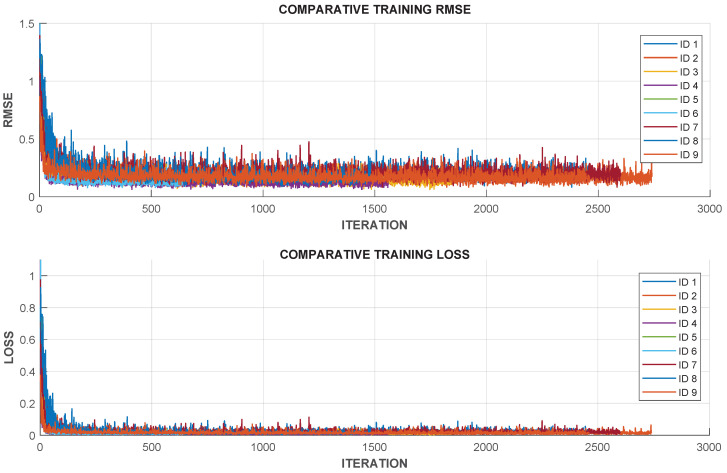
Comparative training RMSE and training loss for different configurations of the LSTM network.

Figure 3 shows the comparative training curves of RMSE and LOSS for the different configurations listed in Table 2. The RMSE curves illustrate how each model adapts to the training data, and the best-performing configurations show lower error values and more stable convergence. The LOSS curves highlight the influence of window size and neuron count on model generalization: shorter windows enabled faster initial convergence but occasionally led to overfitting, while larger windows improved stability at the cost of longer training times. In addition, some configurations (e.g., ID 4, ID 6, and ID 9) displayed noisier convergence and small oscillations, which can be related to the sensitivity of larger models, alternative optimizers, or different activation functions. Such behavior is consistent with known challenges in training recurrent networks, including vanishing or unstable gradients when modeling long temporal dependencies. To mitigate these effects, several stabilization strategies can be considered, such as gradient clipping to control exploding gradients, layer normalization to improve training consistency, and adaptive learning rate schedules to reduce oscillations during optimization. These approaches could improve the robustness of LSTM training.

Based on the results in Table 2 and Figure 3, it is important to highlight the behavior of the tested configurations. The larger window and higher neuron settings (e.g., ID 4 with WS=100,N=100) did not provide superior performance, indicating that excessively long temporal dependencies and larger networks may increase computational cost without improving accuracy. However, smaller windows with more neurons (ID 6) achieved competitive results, suggesting that fine-grained temporal patterns can still be captured effectively. The stacked LSTM configuration (ID 7) also showed good performance but did not surpass the simpler single-layer architecture, demonstrating that additional depth does not always guarantee better results for this application. Finally, variants with a different activation function (ReLU, ID 8) and an alternative optimizer (RMSProp, ID 9) demonstrated stable but slightly lower accuracy compared to the tanh/Adam baseline, confirming that the standard combination remains more suitable for this modeling task. Taking these observations into account, configuration ID 2 (WS=20, N=30, BS=16, VF=20, VP=20) was selected as the final model due to its superior performance in both validation and test metrics, as well as the best balance between training speed, accuracy, and generalization.

**Figure 4:**
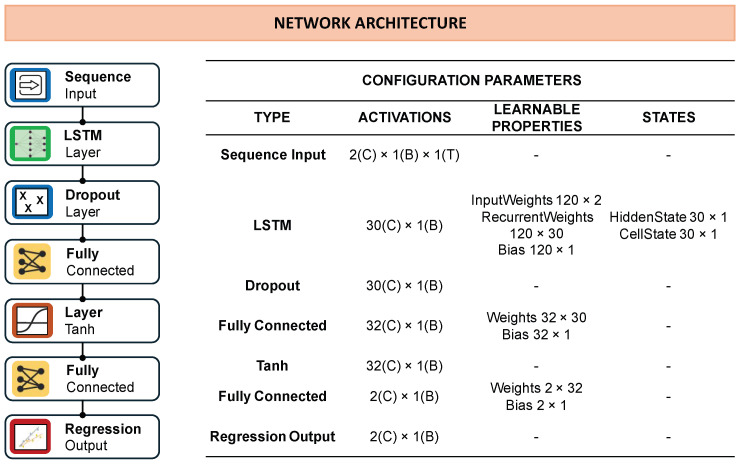
Final LSTM network architecture with details of activation dimensions, learnable properties, and internal states.

The final LSTM model selected is illustrated in Figure 4. With the chosen configuration, in which the window size (*WS*) was set to 20 samples, the total number of sequences in the data set was 16,272 (20 samples fewer than in the SSM model), comprising 12,204 for training, 2441 for validation, and 1627 for testing. This configuration provided an effective balance between complexity and performance, ensuring accurate velocity predictions while maintaining computational efficiency.

### 3.6. Odometry Calculation from Predictions of SSM and LSTM Models

Robot odometry is reconstructed from predicted velocities (*v*, ω) using two complementary kinematic formulations. For linear motion or small angular velocities, the displacement during a sampling interval Δt can be approximated by the following:

(16) $$\left[\begin{array}{c} \Delta x_{t}\\ \Delta y_{t}\\ \Delta \theta_{t} \end{array}\right]=\left[\begin{array}{c} v\cdot \cos\theta \cdot \Delta t\\ v\cdot \sin\theta \cdot \Delta t\\ \omega \cdot \Delta t \end{array}\right]$$

**Figure 5:**
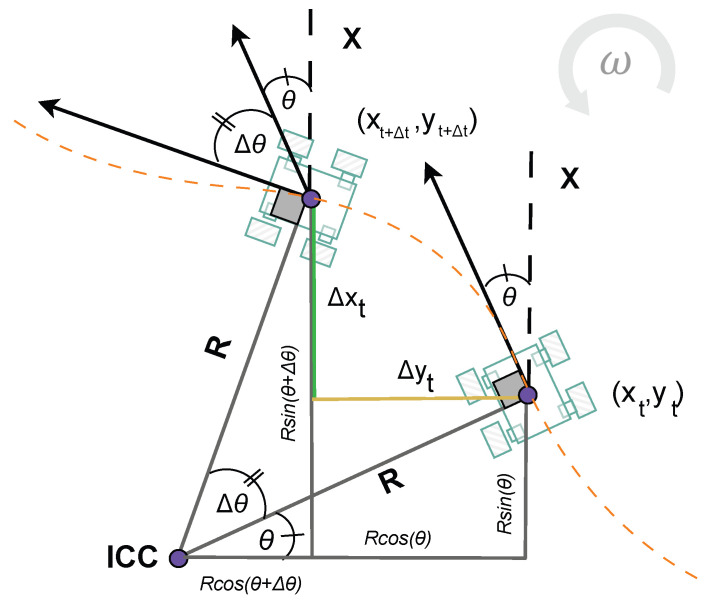
Relationships of circular motion for a robot moving in a trajectory with radius *R* and center in the ICC.

Although this approximation is suitable for straight trajectories, its accuracy decreases in curved motion. To better account for turning maneuvers, the robot’s trajectory is modeled using the concept of the Instantaneous Center of Curvature (ICC), detailed in Figure 5, where the turning radius *R* defines the circular path. In this case, the displacement during Δt is expressed as follows:

(17) $$\left[\begin{array}{c} \Delta x_{t}\\ \Delta y_{t}\\ \Delta \theta_{t} \end{array}\right]=\left[\begin{array}{c} R\cdot \left(\sin(\theta +\omega \cdot \Delta t)-\sin(\theta )\right)\\ -R\cdot \left(\cos(\theta +\omega \cdot \Delta t)-\cos(\theta )\right)\\ \omega \cdot \Delta t \end{array}\right]$$

with the turning radius defined as follows:

(18) $$R=\frac{v}{\omega }$$

Finally, the global pose of the robot can be updated by accumulating the increments:

(19) $$\left[\begin{array}{c} x_{t+\Delta t}\\ y_{t+\Delta t}\\ \theta_{t+\Delta t} \end{array}\right]=\left[\begin{array}{c} x_{t}\\ y_{t}\\ \theta_{t} \end{array}\right]+\left[\begin{array}{c} \Delta x_{t}\\ \Delta y_{t}\\ \Delta \theta_{t} \end{array}\right]$$

where $\left(x_{t},y_{t},\theta_{t}\right)$ denotes the current pose of the robot. This formulation, which combines updates to linear and circular motions, is standard for differential drive robots and has also been presented in our previous work [19].

## 4. Results

The results obtained from the comparison of the SSM and LSTM models are summarized in Table 3 and Table 4. These tables show the results for Experiments 1 and 2, and the corresponding average. The comparison focuses on two aspects: (1) the accuracy in estimating the robot’s linear (*v*) and angular velocity (ω); (2) the odometry estimation based on the predicted velocities.

Specifically, Table 3 shows the FIT and RMSE values when predicting the velocity of each model. The FIT metric explains how well the models fit the actual linear (*v*) and angular (ω) velocities of the robot during the experiments. The RMSE represents the error metric of the difference between the models and the actual measurement. As can be analyzed in Table 3, the SSM model reaches the better FIT, with an average value of 94.70% and 91.71% for *v* and ω, respectively. The average RMSE metric for *v* is similar in both models, with a slightly higher value for the LSTM model in Experiment 1. When analyzing the RMSE value for ω, a slightly better performance of the SSM model is observed, with 0.05 rad/s for SSM versus 0.06 rad/s for LSTM.

Table 4 presents the results of the odometry estimation errors. In Experiment 1, the SSM model achieves lower RMSE values in both position (0.92 m vs. 1.26 m) and angle (0.17 rad vs. 0.27 rad) compared to the LSTM. Similarly, in Experiment 2, the SSM remains more accurate, although the performance gap narrows.

A key observation is that, despite the superior velocity estimation performance of the SSM model, its impact on odometry varies between experiments. In Experiment 1, SSM leads to significantly better odometry accuracy, while in Experiment 2, the differences are less pronounced. This suggests that the characteristics of the trajectory and the accumulated errors influence the overall precision of the odometry.

**Figure 6:**
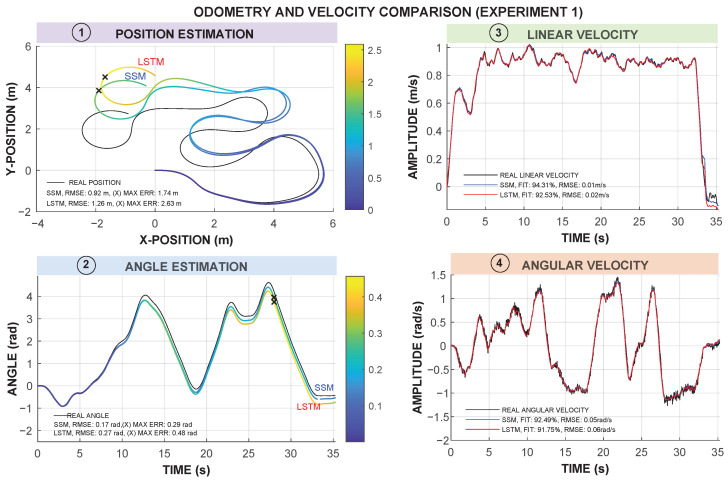
Odometry and velocity comparison in Experiment 1. Subfigures 1 and 2 use color maps to represent position errors (m) and angle errors (rad). “X” markers represent the maximum deviations. Subfigures 3 and 4 present linear and angular velocity comparisons, SSM predictions in blue, and LSTM predictions in red. The actual measurement is shown in black.

**Figure 7:**
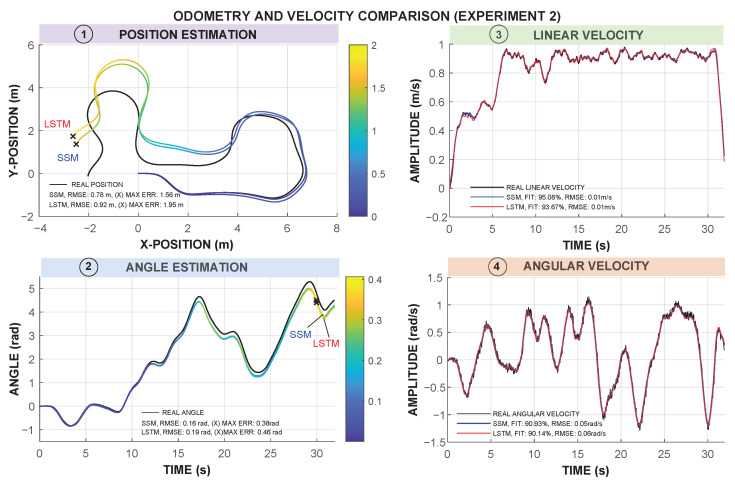
Odometry and velocity comparison in Experiment 2. Subfigures 1 and 2 use color maps to represent position errors (m) and angle errors (rad). “X” markers represent the maximum deviations. Subfigures 3 and 4 present linear and angular velocity comparisons, SSM predictions in blue, and LSTM predictions in red. The actual measurement is shown in black.

Figure 6 and Figure 7 present a comparison of the odometry trajectories estimated by both models for Experiments 1 and 2, respectively. The SSM model consistently provides a trajectory closer to the actual path, particularly in Experiment 1, where deviations in the LSTM model lead to higher cumulative errors. This aligns with the RMSE values in Table 4, confirming that the SSM model yields a more accurate representation of the robot odometry in both position and angle.

In Experiment 1, the LSTM trajectory exhibits a noticeably higher position divergence from the ground truth, especially in sections with sharp turns. This suggests that while the LSTM model could capture non-linearities, it may struggle with sharp transitions. In contrast, Experiment 2 shows a more comparable position performance between both models, reinforcing the idea that trajectory complexity affects model accuracy differently between experiments.

Angle estimation in the SSM model also provides a slightly more accurate result, particularly in Experiment 1. Furthermore, in terms of velocity prediction, the SSM model provides a smoother and more stable response, in linear and angular velocities, better matching the real values.

In addition to prediction accuracy and odometry estimation, the computational performance of both models was assessed by running 100 simulations for each. Table 5 presents the average simulation time, time per integration step, and memory usage for the SSM and LSTM models. The values in Table 5 were obtained by running 100 simulation runs, each consisting of 1577 time steps. The reported simulation time corresponds to the total time required to simulate the entire sequence, while the average time per step was calculated by dividing the total simulation time by the number of steps (1577). Memory usage values represent the amount of memory consumed to simulate the entire sequence. Additionally, for a more granular comparison, the memory usage per step was calculated by dividing the total memory usage by the number of steps. The results clearly show that the SSM model requires significantly fewer computational resources across all metrics. On average, SSM requires 0.00257 ms and 1.03 bytes per simulation step, whereas the LSTM model demands approximately 0.0342 ms and 20.49 bytes per step. These differences highlight the lightweight nature of the classical SSM approach compared to the more computationally intensive LSTM network.

## 5. Discussion

The comparison between the SSM and LSTM models in this study reveals interesting insights into velocity estimation and odometry accuracy. The results indicate that the SSM consistently provides better accuracy in both velocity estimation and trajectory tracking. These results can be further placed in context by comparing them with recent research on mobile robot modeling and control. Some of those papers considered similar modeling paradigms, specifically employing state-space models and those based on deep learning methods to achieve trajectory tracking and velocity estimation. In one work comparing model predictive control (MPC) versus deep neural network-based MPC (DNN-MPC), DNN-MPC had lower position estimation errors, with an error of 123 mm in the *x*-direction and 32 mm in the *y*-direction, compared to 156 mm and 41 mm in traditional MPC [20]. Our work does not compare to this study in a direct sense, but the study implies that accuracy in trajectory tracking can be improved through the use of models based on deep learning, which aligns with the moderate performance of LSTM in our odometry estimation.

Furthermore, the LSTM model in our work had slightly higher RMSE values compared to SSM, specifically in the estimation of the angular velocity (0.06 rad/s for LSTM vs. 0.05 rad/s for SSM). The results of LSTM are in agreement with another work in which an LSTM-based model, with 1000 data groups, had a nearly zero final test error [17]. However, it should be noted that our LSTM model did not achieve the same degree of accuracy as [17], possibly due to differences in the size of the data set, hyperparameter optimization, or noise in robot movement in the real world during our experiments.

Furthermore, our results align with those presented in the study by Farina et al. [18], where the LSTM-based odometry model achieved an accuracy of 86.57%, with RMSE values of 0.102 m/s for linear velocity (v) and 0.179 rad/s for angular velocity (ω). In their study, the traditional odometry method showed a lower global accuracy of 80.2%, with RMSE values of 0.123 m/s for *v* and 0.200 rad/s for ω. However, our experiments demonstrate that the SSM outperformed the LSTM model in both velocity estimation and odometry. Particularly, in Experiment 1, the SSM model had a FIT of 94.31% for *v* and 92.49% for ω, with an RMSE of 0.01 m/s for *v* and 0.05 rad/s for ω, outperforming the LSTM model, where FIT values are 92.53% for *v* and 91.75% for ω, and an RMSE slightly greater, at 0.02 m/s for *v* and 0.06 rad/s for ω. What differentiates our study from that of Farina et al. [18] is the structure of the model. Whereas Farina et al. employed two distinct LSTM networks in parallel to independently estimate the linear and angular velocities of the sensors, our strategy benefits from a multivariate model in which both LSTM and SSM models learn the coupling of *v* and ω. This multivariate framework enables our models to learn the coupling dynamics of both velocities in a single model, thereby having a more holistic and integrated description of the system’s behavior. In addition, stabilization times in previous studies for deep learning-based controllers were reported to be around 2.3 s, with maximum angular errors below 6 degrees [12]. Although our experiments did not explicitly evaluate the stabilization time, the smoother response of the SSM model for *v* and ω suggests that it may provide better stability in real-time applications.

It is also worth mentioning that discrepancies between the estimated and actual trajectories were observed in our study. Such deviations may appear in both the SSM and the LSTM predictions, as small errors can accumulate over time and influence the resulting trajectories. One reason for this discrepancy may be the slippage of the wheel. Recent literature further supports the importance of explicitly accounting for wheel slip and surface-dependent dynamics in off-road mobile robot odometry. In particular, Teji et al. [21] provide a survey of slippage estimation, robot sensing, and control technologies for off-road robots, emphasizing that many state-of-the-art systems still rely on assumptions of negligible slippage despite challenging terrain conditions. Nourizadeh et al. [22,23] proposed in situ methods to estimate slip and skid using proprioceptive sensors, demonstrating how terrain conditions can introduce systematic errors if not adequately accounted for. Similarly, Botta et al. [24] present an estimator of the robot kinematic behavior that explicitly compensates for lateral wheel slip in trajectory planning for mobile robots, showing that slip compensation via kinematic adjustment can significantly reduce trajectory errors. These studies suggest that future extensions of our work should incorporate slip estimation or sliding-velocity modeling, in addition to kinematic constraints, to further improve the robustness of trajectory tracking under realistic conditions.

Finally, the computational cost comparison demonstrates a fundamental trade-off with higher performance of the SSM, outperforming the LSTM in terms of simulation speed and memory consumption. This implies that SSM models might be a better choice in real-time applications or when implemented on low-resource platforms. However, although the SSM model proved to be more efficient in terms of computational performance and accuracy, an advantage observed in the LSTM approach is its flexibility to improve accuracy through hyperparameter tuning. By testing different configurations (e.g., window size, number of neurons, activation functions, and optimizers shown in ), the LSTM model demonstrated variable performance, which in some cases approached that of the SSM. This adaptability indicates that LSTM networks can be adjusted to better capture the dynamics of the system, whereas SSM models are more constrained by their fixed order structure. Therefore, it cannot be ruled out that with more precise tuning, the learning performance of LSTMs could outperform the SSM and become useful in more dynamic or unstructured environments. This characteristic is relevant in engineering practice, as it indicates that neural networks can be adapted to different robotic platforms or operating conditions without requiring a complete reformulation of the mathematical model, as is the case with SSM.

These results encourage further research into hybrid approaches that leverage the strengths of either platform and combine the efficiency of physics-based methods with the flexibility of data-driven approaches.

## 6. Conclusions and Future Work

This study compared the performance of two modeling approaches based on the identification of experimental models. A classical SSM modeling approach was compared with a more contemporary one based on an LSTM network. A second-order model was chosen for the SSM, while a 7-layer architecture was chosen for the LSTM network. The results showed the better performance of the SSM model for linear and angular velocity prediction (FIT of 94.70% for *v* and 91.71% for ω) and for the estimation of odometry (RMSE of 0.85 m and 0.17 rad). The analysis of computational efficiency reveals that the SSM model is significantly more resource efficient, requiring 0.00257 ms and 1.03 bytes per simulation step, while the LSTM model requires approximately 0.0342 ms and 20.49 bytes per step. Although state-space modeling provided a structured and computationally efficient approach to system identification, the LSTM approach demonstrated adaptability through hyperparameter tuning, which allowed performance to approach that of the SSM in some configurations. This flexibility suggests that with more precise tuning and in dynamic or unstructured environments, LSTM models could outperform SSM and become a valuable tool in the identification of robotic systems. The results suggest that for applications that require high accuracy odometry estimation with low computational resources, SSM-based methods remain the most viable choice, whereas LSTM networks represent a promising alternative for future research.

Future research will focus on integrating these models into control frameworks, where robustness can be evaluated under external perturbations such as moving obstacles, varying surface conditions, and wheel slip. In this context, explicit slip estimation or sliding-velocity modeling, in combination with kinematic constraints, may provide a valuable means to improve trajectory tracking under realistic operating conditions. Furthermore, hybrid strategies that combine state-space modeling with neural networks will be explored as a potential pathway to enhance both accuracy and generalization in the identification of robotic systems. In general, this study contributes to the growing body of research on multivariate modeling for mobile robots, reinforcing the complementary roles of classical and modern approaches in the identification of robotic systems and the precision of navigation.

## Figures and Tables

**Table 1:** Testing different SSM orders.

Order	TEST			
	FIT		RMSE	
	*v* (%)	ω (%)	*v* (m/s)	ω (rad/s)
2	97.25	87.72	0.03	0.07
3	97.43	87.79	0.03	0.07
4	97.42	88.04	0.03	0.07
5	97.41	85.23	0.03	0.09

**Table 2:** Different configurations of hyperparameters and the corresponding results.

ID	*ARCH*	*N*	*ACT*	*OPT*	*WS*	*BS*	*VF*	*VP*	Validation				Test			
									FIT		RMSE		FIT		RMSE	
									*v* (%)	ω (%)	*v* (m/s)	ω (rad/s)	*v* (%)	ω (%)	*v* (m/s)	ω (rad/s)
1	LSTM (1)	20	tanh	Adam	10	16	20	20	92.53	89.59	0.06	0.08	92.39	90.09	0.06	0.08
2	LSTM (1)	30	tanh	Adam	20	16	20	20	95.49	90.25	0.04	0.07	95.40	90.36	0.04	0.08
3	LSTM (1)	50	tanh	Adam	50	16	20	20	90.92	91.54	0.08	0.06	90.72	91.75	0.08	0.07
4	LSTM (1)	100	tanh	Adam	100	16	20	20	93.16	89.54	0.06	0.08	93.19	90.03	0.06	0.08
5	LSTM (1)	30	tanh	Adam	70	32	10	10	95.35	90.82	0.04	0.07	94.99	91.25	0.04	0.07
6	LSTM (1)	100	tanh	Adam	30	32	10	20	95.20	90.47	0.04	0.07	94.94	90.13	0.04	0.08
7	LSTM (2)	30	tanh	Adam	20	16	20	30	95.28	90.12	0.04	0.08	95.33	89.51	0.04	0.08
8	LSTM (1)	30	ReLU	Adam	20	16	20	20	94.36	90.73	0.05	0.07	94.39	90.79	0.05	0.07
9	LSTM (1)	30	tanh	RMSProp	20	16	20	20	93.62	88.98	0.05	0.09	93.68	90.32	0.06	0.08

Abbreviations: *WS* (window size), *N* (neurons), *BS* (batch size), *VF* (validation frequency), *VP* (validation patience), *ARCH* (architecture, number of LSTM layers), *ACT* (activation function), *OPT* (optimizer). Metrics reported: Goodness of Fit (FIT, %) and Root Mean Squared Error (RMSE).

**Table 3:** Experimental velocity estimation.

		Linear Velocity		Angular Velocity	
		v (m/s)		ω (rad/s)	
		SSM	LSTM	SSM	LSTM
Experiment 1	FIT (%)	94.31	92.53	92.49	91.75
	RMSE	0.01	0.02	0.05	0.06
Experiment 2	FIT (%)	95.08	93.67	90.93	90.14
	RMSE	0.01	0.01	0.05	0.06
Average	FIT (%)	94.70	93.10	91.71	90.95
	RMSE	0.01	0.01	0.05	0.06

**Table 4:** Experimental odometry estimation.

		Position Estimation		Angle Estimation	
		xy (m)		θ (rad/s)	
		SSM	LSTM	SSM	LSTM
Experiment 1	RMSE	0.92	1.26	0.17	0.27
	Mean	0.74	0.95	0.15	0.23
	STD	0.55	0.83	0.08	0.15
	Max. Error	1.74	2.63	0.29	0.48
Experiment 2	RMSE	0.78	0.92	0.16	0.19
	Mean	0.59	0.66	0.13	0.14
	STD	0.51	0.65	0.10	0.12
	Max. Error	1.56	1.95	0.38	0.46
Average	RMSE	0.85	1.09	0.17	0.23
	Mean	0.67	0.80	0.14	0.19
	STD	0.53	0.74	0.09	0.13
	Max. Error	1.65	2.29	0.33	0.47

**Table 5:** Simulation performance comparison between SSM and LSTM (100 runs).

Metric	SSM	LSTM	Difference (LSTM-SSM)
Sim Time Mean (ms)	4.05	53.89	49.84
Sim Time Std (ms)	11.58	67.28	55.70
Avg Time/Step Mean (ms)	0.00257	0.0342	0.03163
Avg Time/Step Std (ms)	0.00734	0.0427	0.03536
Mem Usage Mean (MB)	0.00155	0.03082	0.02927
Mem Usage Std (MB)	0.00802	0.29999	0.29197
Mem Usage per Step (bytes)	1.03	20.49	19.46

## Data Availability

Dataset available on request from the authors.
